# A monoclonal antibody against KCNK9 K^+^ channel extracellular domain inhibits tumour growth and metastasis

**DOI:** 10.1038/ncomms10339

**Published:** 2016-02-04

**Authors:** Han Sun, Liqun Luo, Bachchu Lal, Xinrong Ma, Lieping Chen, Christine L. Hann, Amy M. Fulton, Daniel J. Leahy, John Laterra, Min Li

**Affiliations:** 1 Department of Neuroscience, Johns Hopkins University School of Medicine, Baltimore, Maryland 21205, USA; 2 Hugo W. Moser Research Institute at Kennedy Krieger, Baltimore, Maryland 21205, USA; 3 Immunotherapy Institute, Fujian Medical University, Fujian 350108, China; 4 Department of Pathology, University of Maryland, Baltimore, Maryland 21201, USA; 5 Department of Immunobiology and Yale Comprehensive Cancer Center, Yale University School of Medicine, New Haven, Connecticut 06511, USA; 6 Department of Oncology, Johns Hopkins University School of Medicine, Baltimore, Maryland 21205, USA; 7 Baltimore Veterans Administration Medical Center, Baltimore, Maryland 21201, USA; 8 Department of Biophysics and Biophysical Chemistry, Johns Hopkins University School of Medicine, Baltimore, Maryland 21205, USA; 9 Department of Neurology, Johns Hopkins University School of Medicine, Baltimore, Maryland 21205, USA

## Abstract

Two-pore domain potassium (K2P) channels act to maintain cell resting membrane potential—a prerequisite for many biological processes. KCNK9, a member of K2P family, is implicated in cancer, owing to its overexpression in human tumours and its ability to promote neoplastic cell survival and growth. However, KCNK9's underlying contributions to malignancy remain elusive due to the absence of specific modulators. Here we describe the development of monoclonal antibodies against the KCNK9 extracellular domain and their functional effects. We show that one antibody (Y4) with the highest affinity binding induces channel internalization. The addition of Y4 to KCNK9-expressing carcinoma cells reduces cell viability and increases cell death. Systemic administration of Y4 effectively inhibits growth of human lung cancer xenografts and murine breast cancer metastasis in mice. Evidence for Y4-mediated carcinoma cell autonomous and immune-dependent cytotoxicity is presented. Our study reveals that antibody-based KCNK9 targeting is a promising therapeutic strategy in KCNK9-expressing malignancies.

## Abstract (web summary)

The potassium channel KCNK9 mediates important biological processes and is often overexpressed in breast and lung cancers. In this study, the authors developed a specific monoclonal antibody against the extracellular domain of KCNK9 and show that it inhibits cancer growth and metastasis *in vivo* through both cell autonomous and immune-dependent cellular cytotoxicity.

## Graphical abstract

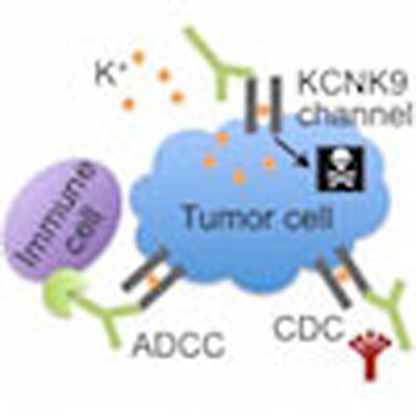

Ion channels facilitate the passage of ions across cellular membranes in all organisms. Transient change of ionic distribution alters membrane potential, which forms the basis for a variety of biological processes. Potassium (K^+^) channels are the most abundant and diverse ion channels^1^. Among them, two-pore domain K^+^ (K2P) channels are the newest members. To date, 15 mammalian K2P channel subtypes (Fig. 1a) have been discovered^2^ and each subtype plays a distinct role in physiological processes and disease, including mental retardation, familial migraine and cancer^2^,^3^,^4^,^5^. Despite their significance, we have gained limited knowledge about individual K2P subtypes partly due to K2Ps' nature of being highly homologous and the paucity of subtype-specific tools.

KCNK9 is a member of the K2P channel family. Under physiological conditions, KCNK9 is primarily expressed in tissues of the central nervous system such as the cerebellum and acts to maintain resting membrane potential and regulate action potential firing^2^. KCNK9 has also been implicated in cancer based on genetic evidence. For instance, 10% of breast tumours showed 3- to 10-fold *KCNK9* genomic amplification, along with 5-fold to over 100-fold messenger RNA overexpression in 40% of breast and lung cancers^5^. Enforced KCNK9 expression promotes malignant transformation of mouse mammary gland epithelial cells and embryonic fibroblasts in nude mice, possibly by improving cell survival under hypoxic or serum-deprived conditions^5^,^6^. However, how endogenous KCNK9 contributes to neoplasia and its potential as a therapeutic target remain elusive due to the lack of specific modulators of KCNK9 functions. Genetic studies of K2P channels are often difficult to interpret because of developmental and compensatory effects^7^. High-throughput chemical screening has been carried out to identify KCNK9-specific probes but has resulted in limited progress^8^. This is partly because it is difficult to design chemical screens for targets with high sequence and structure homology. Antibodies, known for their exquisite selectivity, have been broadly used to target cell surface receptors and antigens, especially as applied to cancer treatment^9^,^10^,^11^. However, the feasibility of using antibodies to modulate ion channel activity is not well explored.

K2P channels share considerable architectural similarity. They assemble as dimers; each subunit contains two pore-lining regions (P1 and P2) and four transmembrane domains (M1–M4). One signature feature of K2P channels is a loop of ∼60 amino acids on the extracellular side between the M1 and P1 domains, known as the M1P1 loop. Crystal structure analysis of human K2P channels reveals this loop as a structured domain that ‘caps' the extracellular ion pathway, providing an explanation for K2P's insensitivity to common channel blockers^12^,^13^. Mutational analysis and chimera studies have provided compelling evidence for M1P1 loop's role in sensing extracellular stimuli and regulating channel gating^14^,^15^. Sequences within the M1P1 loop are poorly conserved among K2P subtypes, representing a desirable extracellular epitope reservoir. Provided that the M1P1 loop harbours important modulatory sites^14^,^15^, we hypothesize that antibodies raised against the M1P1 loop will allow selective manipulation of KCNK9 functions.

In this study, we developed an inhibitory antibody against the extracellular domain of KCNK9. We characterized antibody-based KCNK9 targeting and found it effectively inhibited cancer cell survival, tumour growth and metastasis. Knowledge and research strategies gained from this study are likely to have general benefits to studies of other related channels in health and disease.

## Results

### M1P1 targeting antibodies inhibit KCNK9 channel activity

**Figure 1:**
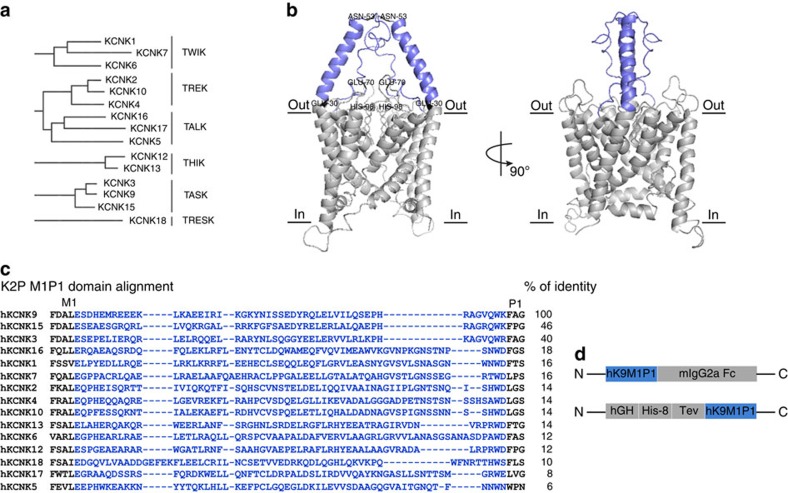
Characteristics of the target and antigens. (**a**) K2P phylogenetic tree calculated from a sequence alignment of the 15 human channel pore domain 1 sequences. K2P channels can be divided into six subfamilies based on sequence similarity and functional resemblance. (**b**) Three-dimensional structure of hKCNK9 predicted by homology modelling based on hKCNK4 crystal structure. The M1P1 loop targeted by antibodies is coloured blue. Residues within M1P1 loop essential for gating or posttranslational modification were annotated. (**c**) Sequence alignment corresponding to the M1P1 loop of K2P subtypes. The M1P1 loop chosen for antibody generation is coloured blue. Pairwise percentage of identical residues between the M1P1 loop of hKCNK9 and other K2P subtypes was calculated. (**d**) Schematic representation of recombinant antigens: hK9M1P1-mIgG and hGH-hK9M1P1.

**Figure 2:**
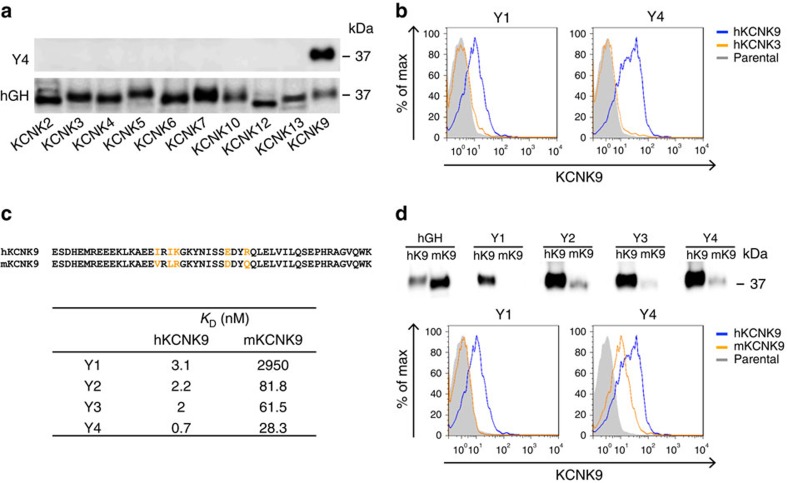
Y4 binds to hKCNK9 with subtype specificity and high affinity. (**a**) Western blot analysis of hGH-hK2PM1P1 recombinant proteins blotted with Y4 and anti-hGH antibodies. Uncropped blots are shown in Supplementary Fig. 2b. (**b**) Flow cytometry analysis of HEK293 cells transiently expressing hKCNK9 or hKCNK3. (**c**) Sequence alignment corresponding to the M1P1 loop of hKCNK9 and mKCNK9 with non-conserved residues highlighted (top). Binding kinetics of Y-mAbs determined by Octet platform (bottom). (**d**) Y-mAbs' differential affinity to hKCNK9 versus mKCNK9 validated by western blotting of recombinant proteins and flow cytometry analysis of HEK293 cells transiently expressing hKCNK9 or mKCNK9. Uncropped blots are shown in Supplementary Fig. 2c.

To generate antigens that recapitulate native human KCNK9 (hKCNK9) structure, the M1P1 loop (Fig. 1b,c) was expressed as a recombinant protein in HEK293T and CHO-S cells, to optimize preservation of three-dimensional structure and posttranslational modifications (Fig. 1d and Supplementary Fig. 1). Forty murine monoclonal antibodies were successfully generated. Among them, 4 monoclonal antibodies were raised to hK9M1P1-mIgG2aFc, designated as Y-mAbs; 36 monoclonal antibodies were raised to hGH-hK9M1P1, designated as H-mAbs. All monoclonal antibodies were IgG1 and demonstrated a subtype-specific binding to hKCNK9 over other K2P subtypes including hKCNK3—the K2P member most closely related to hKCNK9 (Fig. 2a,b and Supplementary Fig. 2). Y-mAbs displayed nanomolar to subnanomolar affinity, to recombinant hKCNK9 protein with Y4 having the highest affinity (*K*_D_∼0.7 nM). Y-mAbs also cross-reacted with recombinant murine KCNK9 (mKCNK9) with a lower affinity (Fig. 2c,d). The degree of cross-reactivity was different among the Y-mAbs. As hKCNK9 and mKCNK9 M1P1 loops differ by only five amino acids (Fig.2c), differential cross-reactivity provides insight into the distinctive epitopes recognized by the Y-mAbs.

**Figure 3:**
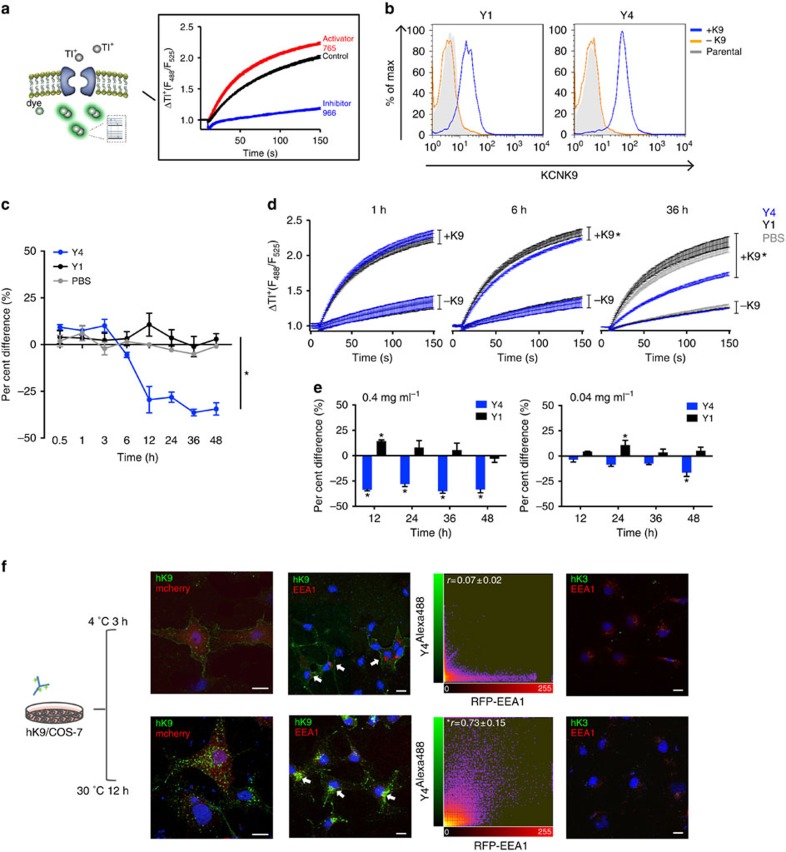
Y4 inhibits hKCNK9 by inducing internalization of channel from cell surface. (**a**) Schematic representation of Tl^+^-based fluorescence assay principle (FluxOR Potassium Ion Channel Assay, Invitrogen) and readout. Compound 765 and 966 were chemical modulators used as control compounds. (**b**) Flow cytometry analysis of tetracycline-inducible hKCNK9 stable cell line stained with Y1 and Y4 in the presence of tetracycline induction, designated as +K9 or in the absence of tetracycline induction, designated as −K9. Parental HEK293 cells were used as negative control. (**c**) Time-dependent inhibition of Tl+ conductance by Y4. Tetracycline-induced cells were treated with Y4, Y1 or PBS before Tl^+^ assay and compared with cells without treatment. Difference in Tl^+^ conductance at time point 80 was used for comparison. Mean±s.d., *n=2*4 (the experiment was replicated four times), *P<0.0001, one-way analysis of variance (ANOVA). (**d**) Representative Tl^+^ traces from tetracycline-induced KCNK9-expressing cells (+K9) and non-induced cells (−K9) plotted as fluorescence change over time. Mean±s.d., *n=24* (the experiment was replicated four times), *P<0.01, two-tailed Student's *t*-test. (**e**) Concentration-dependent inhibition of Tl^+^ conductance by Y4. Mean±s.d., *n=*6 (the experiment was replicated twice), *P<0.01, two-tailed Student's *t*-test. (**f**) Internalization of hKCNK9 from cell surface induced by Y4. COS-7 cells transiently expressing hKCNK9 and mcherry or red fluorescent protein (RFP)-tagged EEA1 were incubated with Alexa488-conjugated Y4. Cells transiently expressing hKCNK3 and RFP-tagged EEA1 were control. Nuclei were counterstained with 4,6-diamidino-2-phenylindole (DAPI). Co-localization (arrows) between Alexa488-conjugated Y4 and RFP-tagged EEA1 was analysed using Imaris software and representative scatter plots were shown. Statistical analysis was based on Pearson's correlation coefficient calculated from 20 fields, mean±s.d. *r>0.5. Scale bar, 20 μm.

We tested the effects of Y- and H-mAbs on hKCNK9 channel using a thallium (Tl^+^)-based fluorescent ion-flux assay developed for hKCNK9 chemical screening^8^,^16^. In this assay, recorded fluorescent signal is proportional to the number of open K^+^ channels on the cell surface (Fig. 3a). Twelve monoclonal antibodies showed positive staining on the surface of hKCNK9-expressing HEK293 cells and their effects on hKCNK9 function were assessed in the Tl^+^ assay (Fig. 3b and Supplementary Fig. 3a). Six of these monoclonal antibodies were effective in inhibiting hKCNK9 conductance following a preincubation protocol (Fig. 3c and Supplementary Fig. 3b,d). This inhibition was not due to differences in cell viability as indicated by comparable fluorescence intensities at time 0 of fluorescence measurement (Supplementary Fig. 3c). Y4 was most potent showing up to 30% inhibition of Tl^+^ signal after 36 h monoclonal antibody incubation. Hence, we chose Y4 for follow-up studies. Time-course experiments indicated that Y4-mediated channel inhibition was significant after 6 h incubation. The inhibitory effect was stronger after longer exposures (Fig. 3c,d and Supplementary Fig. 3d) and dependent on the monoclonal antibody concentration (Fig. 3e). We also set up electrophysiological assays to examine the possibility of acute blocking of hKCNK9 by Y4 and found no detectable effects with 20 min of perfusion (Supplementary Fig. 4).

### Y4 induces KCNK9 channel internalization from cell surface

The time dependence of Y4-mediated channel inhibition suggested a mechanism of antibody-induced endocytosis^17^,^18^,^19^ and we examined this possibility using flow cytometry. hKCNK9-overexpressing HEK293 cells were incubated with Y-mAbs for 12 h at 30 °C and channels remaining on the cell surface were stained, as outlined in Supplementary Fig 5a. Flow cytometry analysis showed that 15.6% KCNK9-positive cells completely lost cell surface signal and became KCNK9 negative, and among cells that remained KCNK9-positive their overall fluorescence decreased by 25.7%. Reduction of cell surface KCNK9 occurred only in response to Y4 treatment but not to treatment with other Y-mAbs (Supplementary Fig. 5b–d). For further analysis, we used confocal microscopy to track fluorescently labelled Y4. Fluorescent conjugation of Y4 did not alter its binding affinity (Supplementary Fig. 5h). Upon incubation, a consistent reduction of surface signal along with increased intracellular signal, predominantly visible in the perinuclear region, was observed only at a temperature permissive to endocytosis. Internalized Y4 co-localized with EEA1, an early endosome marker (Fig. 3f). This phenomenon is specific to hKCNK9, as no change was detected in cells expressing hKCNK3 (Fig. 3f). Taken together, these data indicate that monoclonal antibody-induced endocytosis of functional channels from cell surface can be one of the mechanisms that led to reduced channel conductance observed in the ion-influx assay.

### Y4 recognizes endogenous KCNK9 in cancer cell lines

**Figure 4:**
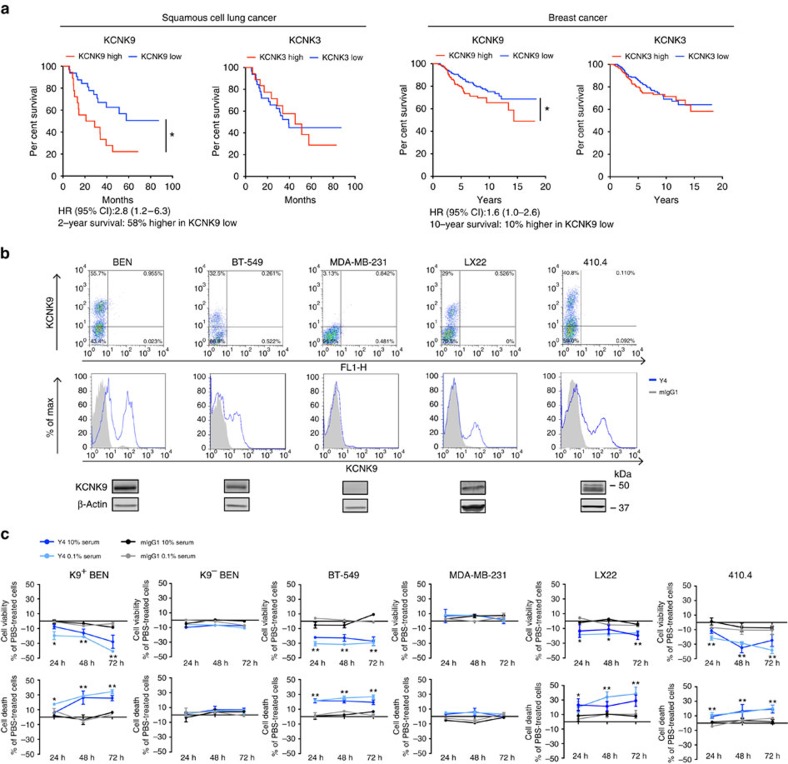
KCNK9 shows clinical relevance and Y4-mediated targeting exhibits anti-cancer effects *in vitro*. (**a**) Kaplan–Meier curves for survival of patients with squamous cell lung cancer and breast cancer in relation to *KCNK9* gene expression level. Data sets were obtained from commonly used published studies^20^,^21^. Patients' gene expression data and clinical data were manually matched. Patients with both *KCNK9* expression and survival data were segregated into two groups according to *KCNK9* expression level—KCNK9 high (twofold higher than mean) and KCNK9 low (twofold lower than mean). Survival was analysed using the Prism Kaplan–Meier package and log-rank Mantel–Cox statistical test. We performed the same analysis to examine *KCNK3* expression versus survival. *n=*53 (lung), *n=*289 (breast), *P<0.05, log-rank Mantel–Cox. (**b**) Y4's ability to detect endogenous KCNK9 expressed in cancer cell lines verified by flow cytometry and western blotting analyses. (**c**) Treatment with Y4 for 24–72 h reduces cell viability and increases cell death of KCNK9-expressing cancer cell lines. BEN cells were sorted according to hKCNK9 expression level (K9^+^ or K9^−^). Mean±s.d., *n=*6, *P<0.01 at 0.1% heat-inactivated (HI) serum, **P<0.01 at 0.1% and 10% HI serum, two-tailed Student's *t*-test.

*KCNK9* overexpression has been reported in ∼30% of both breast and lung cancers^5^. Enforced KCNK9 expression promotes tumour-propagating capacity of non-neoplastic cells and this property is eliminated by co-expressing a dominant-negative KCNK9 mutant, suggesting a role for KCNK9 during tumour growth^5^,^6^. To assess the clinical relevance of KCNK9, correlations between *KCNK9* gene expression and patient survival were analysed using publicly available microarray data sets^20^,^21^. We found an inverse correlation between *KCNK9* expression and overall survival of patients with either squamous cell lung or breast cancer (*P*<0.05). In squamous cell lung cancer, the 2-year relative survival rates of low *KCNK9* expressers increased by 58% compared with high expressers. The hazard ratio between *KCNK9* high and low groups is 2.8 (95% confidence interval: 1.222–6.329). In breast cancer patients, the 10-year relative survival rates of low *KCNK9* expressers increased by 10% compared with high expressers. The hazard ratio between *KCNK9* high and low groups is 1.6 (95% confidence interval: 1.005–2.556). In both cancer types, this survival benefit is *KCNK9* specific, as the same analysis using *KCNK3*, the closest related member of *KCNK9*, showed no significant correlations with patient survival (Fig. 4a). This supports the notion that KCNK9 promotes tumour growth and may be a therapeutic target in KCNK9-expressing malignancies.

Given that Y4 binds and internalizes hKCNK9 in a K2P subtype-specific manner, it affords an advantageous strategy to investigate the role of endogenous KCNK9 in cancer biology. The BEN squamous cell lung cancer cell line was previously shown to have abundant hKCNK9 mRNA^6^. This was confirmed at the protein level and cell surface channel expression was validated by flow cytometry (Fig. 4b). Subsequently, we screened a panel of human and murine cancer cell lines by quantitative reverse transcriptase–PCR (Supplementary Fig. 6a,b) followed by validation via western blotting and flow cytometry. In addition to BEN, the BT-549 human breast cancer cell line and cells from LX22 patient-derived xenograft of small cell lung cancer were found to express hKCNK9 at a relatively high level. The MDA-MB-231 human breast cancer cell line, on the other hand, had undetectable expression of hKCNK9 (Fig. 4b). mKCNK9 was present in metastatic and non-metastatic murine breast cancer cell lines (Supplementary Fig. 6b). Higher expression levels were found in two metastatic cell lines 66.1 and 410.4, suggesting a potential role of KCNK9 in metastasis. Y4 stained 410.4 cells, indicating sufficient cross-reactivity with mKCNK9 (Fig. 4b). Interestingly, BEN, BT-549, LX22 and 410.4 cells showed two cell populations based on surface staining of KCNK9, designated as K9^+^ and K9^−^. K9^−^ BEN cells were stable after *in vitro* culture; hence, we used them as a negative control for subsequent *in vitro* studies (Supplementary Fig. 6c).

### Y4 reduces cell viability and increases cell death

Inhibition of KCNK9 via a dominant-negative mutant or short hairpin RNA was shown to inhibit cancer cell proliferation by 25∼65% (refs 6, 22). Similar to the effect of Y4 on inducing the internalization of transgenic hKCNK9 in HEK293, we found that Y4 induced internalization of endogenous KCNK9 in BEN cell line with 21.7% reduction of KCNK9-positive cells and 9.8% reduction of overall fluorescence in KCNK9-positive cells (Supplementary Fig. 5e–g). To evaluate whether this Y4-mediated modulation of KCNK9 has any biological effects, BEN, BT-549, MDA-MB-231, LX22 and 410.4 cells were treated with antibodies 24–72 h in 10 or 0.1% heat-inactivated serum. Y4 applied at 0.4 mg ml^−1^ significantly suppressed the viability of K9^+^ BEN, BT-549, LX22 and 410.4 cells but not K9^−^ BEN or MDA-MB-231 cells (Fig. 4c). Inhibition of K9^+^ BEN and LX22 cells was more prominent at 0.1% serum, consistent with previous reports showing more pronounced effects of mutant KCNK9 in low serum^6^.

Changes in cell viability could be due to alterations in cell proliferation, cell death or metabolism. To further dissect Y4's biological effects, we measured L-lactate dehydrogenase (LDH) release as a readout of cell death following monoclonal antibody treatment. Y4 induced significant cell death of K9^+^ BEN, BT-549, LX22 and 410.4 cells. No significant LDH release was detected in K9^−^ BEN or MDA-MB-231 cells (Fig. 4c). This suggests that the observed reduction in cell viability could be attributed to increased cell death. The correlation of cytotoxicity with target abundance is consistent with a KCNK9-specific mechanism.

### Y4 suppresses tumour growth *in vivo*

**Figure 5:**
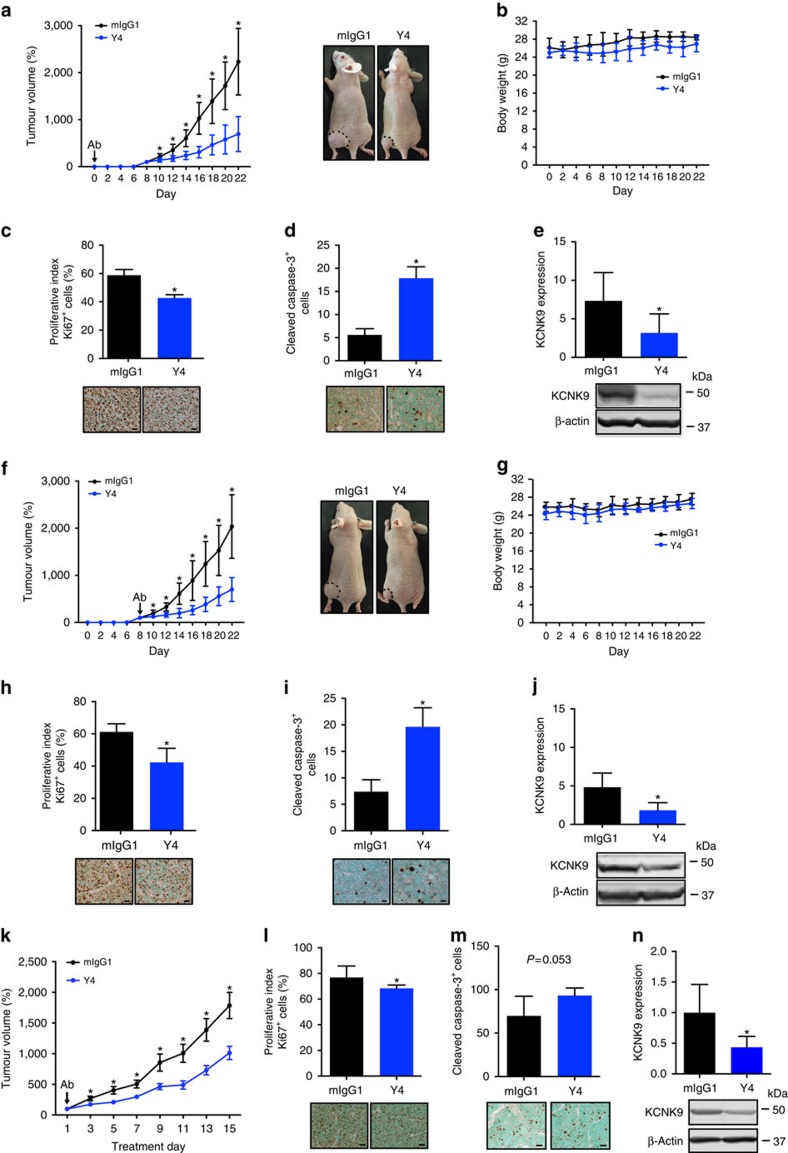
Y4 inhibits the capacity of BEN cells to propagate subcutaneous tumour xenografts. (**a**) Growth curves of BEN engraftments in nu/nu mice treated with mIgG1 or Y4 i.p. twice a week for 22 days. Mean±s.d., *n=*10 per group, *P<0.01, two-tailed Student's *t*-test. mAb treatment started on the same day of cell injection. Representative photographs showing tumours (dotted line) formed on day 22. (**b**) Body weights of mice. Mean±s.d., *n=*10 per group. (**c**) Quantitative determination of Ki67^+^ cells and representative staining of tumour cross-sections. Mean±s.d., *n=*30, *P<0.01, two-tailed Student's *t*-test; scale bar, 1,000 μm. (**d**) Quantitative determination of cleaved caspase-3^+^ cells and staining photographs of tumour cross-sections. Mean±s.d., *n=*30, *P<0.01, two-tailed Student's *t*-test; scale bar, 1,000 μm. (**e**) Quantitative determination of hKCNK9 and representative western blotting of tumour tissues. Mean±s.d., *n=*10, *P<0.05, two-tailed Student's *t*-test. (**f**) Growth curves of BEN engraftments in nu/nu mice treated with mIgG1 or Y4 i.p. twice a week for 14 days. Mean±s.d., *n=*10 per group, *P<0.01, two-tailed Student's *t*-test. mAb treatment started after tumour was measurable on day 8. Representative photographs showing tumours (dotted line) formed on day 22. (**g**) Body weights of mice. Mean±s.d., *n=*10 per group. (**h**) Quantitative determination of Ki67^+^ cells and representative staining of tumour cross-sections. Mean±s.d., *n=*30 per group, *P<0.01, two-tailed Student's *t*–test; scale bar, 1,000 μm. (**i**) Quantitative determination of cleaved caspase-3^+^ cells and representative staining of tumour cross-sections. Mean±s.d., *n=*30 per group, *P<0.01, two-tailed Student's *t*-test; scale bar, 1,000 μm. (**j**) Quantitative determination of hKCNK9 and representative western blotting of tumour tissues. Mean±s.d., *n=*10 per group, *P<0.05, two-tailed Student's *t*-test. (**k**) Growth curves of LX22 patient-derived xenograft (PDX) engraftments in NSG mice treated with mIgG1 or Y4 i.p. twice a week for 15 days. Mean±s.e.m., *n=*10 per group, *P<0.01, two-tailed Student's *t*-test. (**l**) Quantitative determination of Ki67^+^ cells and representative staining of tumour cross-sections. Mean±s.d., *n=*30 per group, *P<0.05, two-tailed Student's *t*-test; scale bar, 1,000 μm. (**m**) Quantitative determination of cleaved caspase-3^+^ cells and representative staining of tumour cross-sections. Mean±s.d., *n=*30 per group; scale bar, 1,000 μm. (**n**) Quantitative determination of hKCNK9 and representative western blotting of tumour tissues. Mean±s.d., *n=*10 per group, *P<0.05, two-tailed Student's *t*-test.

To investigate Y4's therapeutic effects, antibodies were administered intraperitoneally (i.p.) at 4 mg kg^−1^ into nu/nu mice either on the same day of BEN subcutaneous engraftments, to monitor effects during tumour initiation, or after visible tumours formed, to monitor effects on established tumours. Y4 effectively suppressed tumour growth in both experimental designs (Fig. 5a,f). In comparison, another anti-KCNK9 monoclonal antibody (H8) had no effects on tumour growth (Supplementary Fig. 7). Systemic administration of Y4 was well tolerated in mice as suggested by no dramatic changes in body weight (Fig. 5b,g). Ki67 and cleaved caspase-3 staining showed that Y4-treated tumour sections had a lower proliferative index and a higher apoptotic index, respectively (Fig. 5c,d,h,i). Protein extractions from Y4-treated tumours also showed reduced hKCNK9 expression as normalized to β-actin (Fig. 5e,j). We verified Y4's *in vivo* effects in LX22—a highly aggressive patient-derived xenograft model of small cell lung cancer^23^—and found similar results on tumour growth inhibition, reduction of proliferative index and KCNK9 downregulation (Fig. 5k–n). These results indicate that Y4 treatment is able to hinder tumour growth by affecting proliferation and/or apoptosis and it downregulated tumour-associated hKCNK9.

### Y4 suppresses metastasis *in vivo*

**Figure 6:**
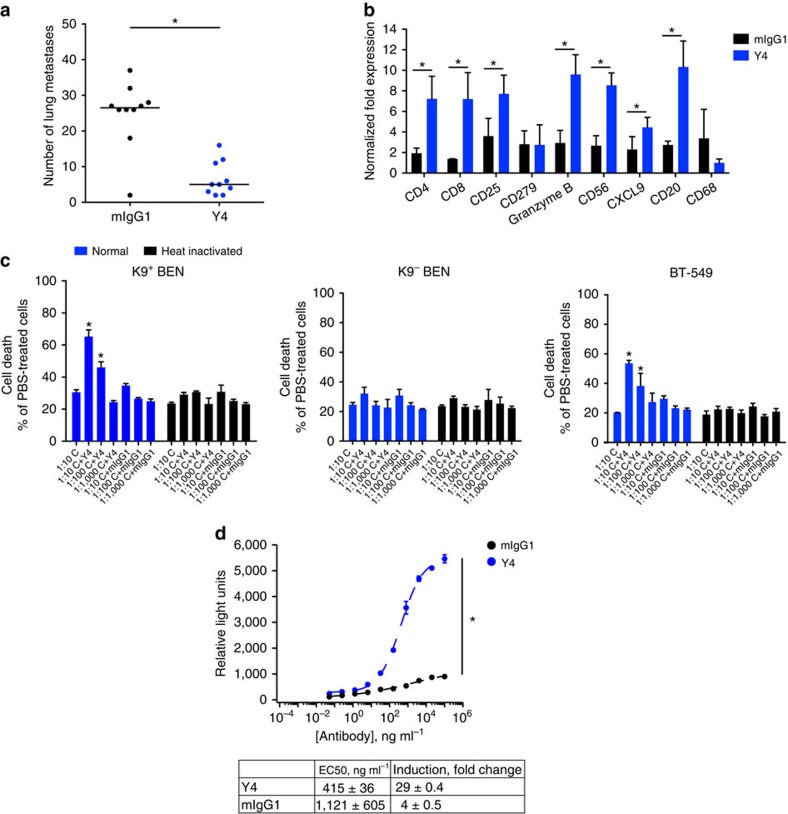
Y4 inhibits lung metastasis and induces immune responses. (**a**) Number of lung metastasis foci in BALBc/ByJ mice treated with mIgG1 or Y4 i.p. twice a week for 25 days. The centre line represents median, *n=*10 per group, *P<0.01, two-tailed Student's *t*-test. (**b**) Expression of immune markers verified by quantitative reverse transcriptase–PCR (qRT–PCR) of mRNAs extracted from lung tissues. Mean±s.d., *n=*5, *P<0.05, two-tailed Student's *t*-test. (**c**) Y4 activates complement, resulting in complement-dependent cytotoxicity *in vitro*. K9^+^ BEN cells and BT-549 cells were treated with Y4 for 2 h in the presence of either normal or heat-inactivated murine complement before LDH measurement. Mean±s.d., *n=*6, *P<0.05, two-tailed Student's *t*-test. (**d**) Y4 activates ADCC reporter cells. BEN cells were incubated with different doses of Y4 and mIgG1, and engineered murine Jurkat T cells stably expressing FCγRIII and an NFAT-response element that drives luciferase expression (NFAT-RE-luc2). Measured luciferase activity indicates ADCC induction. Mean±s.d., *n*=3, *P<0.01, two-tailed Student's *t*-test.

As we found relatively high KCNK9 expression in metastatic murine breast cancer cell lines, we went on to examine Y4's effects on metastasis. The cross-reactivity of Y4 with mKCNK9 provided an opportunity to study metastasis in immunocompetent syngeneic animals. We injected 410.4 cells into the tail vein of BALB/cByJ mice and examined Y4's effects on lung colonization. Y4-treated animals showed significantly fewer lung metastases with no detectable toxicity (Fig. 6a). These data suggest that Y4 not only is effective against primary tumour formation but also metastasis. Y4 is a murine IgG1 isotype, which possesses the ability to mediate effector functions including complement-dependent cytotoxicity (CDC) and antibody-mediated cellular cytotoxicity (ADCC). Thus, inhibition of lung metastasis could be due to direct effects via KCNK9 inhibition and/or elevated immune responses via monoclonal antibody effector functions. We extracted mRNA from lung tissues and characterized the expression of immune markers^24^. Y4-treated mice showed higher infiltration of CD56^+^ natural killer cells, granzyme B^+^ cytotoxic T cells and increased T-cell chemoattractant chemokine (C-X-C motif) ligand-9 (Fig. 6b). We have shown previously that enforced expression of chemokine (C-X-C motif) ligand-9 inhibits growth and metastasis of murine breast cancer cells by a mechanism involving natural killer and T cells^25^.

To explore Y4's potential for inducing immune effector responses, we performed *in vitro* CDC and ADCC assays. We incubated cancer cells with Y4 for 2 h in the presence of murine complement before LDH assay. Y4 was effective in inducing CDC against K9^+^ BEN and BT-549 cells. This response was dependent on the complement concentration, whereas no effects were found in K9^−^ BEN cells or if complement was heat inactivated (Fig. 6c). To investigate the possibility of Y4-induced ADCC, we incubated BEN cells with increasing Y4 concentrations in the presence of bioluminescent Jurkat T-reporter cells established for ADCC studies^26^. We found a dose-dependent induction of ADCC as indicated by luciferase activity (Fig. 6d). Based on these *in vitro* findings, we speculate that Y4-mediated inhibition of metastasis may involve both cell-autonomous effects and/or immune-dependent pathways.

## Discussion

We developed a monoclonal antibody-based strategy that selectively and effectively inhibits the function of KCNK9 via a mechanism that involves monoclonal antibody-induced internalization. Taking advantage of this tool, we discovered KCNK9's pivotal role in promoting cancer cell survival and growth, and that monoclonal antibody-based targeting of KCNK9 has therapeutic promise in the treatment of primary tumour and metastasis, through inhibiting KCNK9 channel function and/or activating anti-tumour immune responses.

The human genome encodes over 400 ion channel genes. This vast number and diversity allow ion channels to mediate a broad spectrum of biological processes including nerve and muscle excitation, hormone secretion, cell proliferation, learning and memory. Consequently, defects in ion channel function often have profound physiological effects. To date, mutations in over 60 different ion channel genes have been found to cause human diseases^27^. Because of their clinical significance, ion channels make up the second largest class of drug targets^28^. Despite extensive investigation, 40% of characterized ion channels have no known ligand/drug reported and most inhibitors for the remaining 60% are nonselective. Therefore, there is an increased focus towards more selective pharmacological agents^27^. Antibodies constitute the fastest growing class of therapeutics. Antibody-based therapies are well established for cancer and inflammatory diseases^11^,^29^,^30^. Although antibodies are effective tools for ion channel research, the utilization of antibodies as ion channel modulators is poorly explored. This is due, in part, to poor epitope accessibility and specificity of extracellular domains, as well as technical challenges in obtaining high-quality antigens and establishing effective functional screening. This is probably why most antibody modulators reported so far are polyclonal with little or no biological activity^1^.

High-resolution mapping of an amplicon in human breast and lung cancers identified *KCNK9* as the only gene with genomic amplifications. Follow-up analysis confirmed KCNK9 overexpression at the mRNA and protein levels^5^. Enforced KCNK9 expression conferred tumorigenicity to otherwise non-tumorigenic cells^5^. Since these initial studies, KCNK9's oncogenic properties have been explored in multiple cancer cell lines^6^,^22^,^31^. Depending on the cancer type and method used to manipulate channel function, KCNK9 had different roles in tumorigenicity. For example, inhibiting KCNK9 via dominant-negative mutant, anti-sense short hairpin RNA or nonspecific chemical blockers suppressed proliferation of lung carcinoma and melanoma cells^6^,^22^. In contrast, pharmacological manipulation of KCNK9 with chemical modulators known to lack subtype specificity led to contradictory results in glioma cell lines^27^. Differences in these *in vitro* studies could be attributed to differences among cancer types or off-target effects of chemical modulators. This further supports the need for highly selective pharmacological agents to investigate KCNK9 function and it highlights the advantage of monoclonal antibody-based targeting. Indeed, the monoclonal antibody reported here affords the first demonstration of the oncogenic properties of endogenous KCNK9 channel *in vivo* and we observed consistent tumour-promoting properties in tumour growth and metastasis.

Numerous K^+^ channels in addition to KCNK9 have been reported to be differentially expressed in human cancer and regulate different aspects of tumorigenecity^32^,^33^. However, it is still largely unknown how K^+^ ion conductance controls biological processes. K^+^ channels, K2P in particular, fine-tune resting membrane potential to keep it at a generally defined range of −30 to −85 mV. As channels open, the membrane potential can either become more negative (hyperpolarize) or become more positive (depolarize), in comparison with the resting membrane potential. Changes in membrane potential can alter cell physiology such as cell volume dynamics, which in turn regulate cell proliferation, adhesion and migration^32^,^33^. For example, calcium (Ca^2+^)-activated K^+^ channels (K_Ca_) and inward rectifying K^+^ channels (K_ir_) can regulate local cell volume to promote cancer cell invasion^31^. In addition, maintenance of resting membrane potential is critical to the function of voltage-sensitive molecules. For example, hyperpolarization caused by opening of K2P channels increases Ca^2+^ influx and regulates G_1_–S transition during cell cycle progression^34^. Membrane potential oscillation may also directly control cell cycle progression. The expression and function of K^+^ channels are known to show cell cycle phase dependence^32^. A number of reports indicate that membrane hyperpolarization accompanied with increased K^+^ permeability takes place at G_1_–S transition and it is required to initiate S phase, whereas depolarization prevents G_1_–S transition^33^,^34^,^35^,^36^. Preliminary cell cycle analysis with BEN cells indicated that KCNK9 expression disproportionally associated with S+G_2_/M phases (Supplementary Fig. 8). As the opening of KCNK9 results in hyperpolarization, it suggests that increased expression/activity of KCNK9 promotes cell cycle progression through G_1_–S. This would support the findings that KCNK9 targeting by Y4 reduced proliferative index and decreased tumour burden *in vivo*. In our study, by comparing Y4 and H8, we found that the degree of KCNK9 conductance inhibition correlates with antibody's anti-cancer effect *in vivo*. In addition, modulation of KCNK9 by Y4 in the absence of immune-dependent factors such as complement is sufficient to cause profound cytotoxicity *in vitro.* These results suggest that KCNK9 is not simply a tumour antigen but an important regulator of tumour growth. Defining the mechanistic network linking K^+^ homeostasis and tumour physiology awaits further studies.

There are multiple mechanisms by which antibodies perturb channel function. Most antibodies generated by rational design either occlude the ion permeation pathway or alter modalities essential for channel gating and their effects often become apparent within minutes of antibody addition^1^,^37^. Antibody-induced internalization has been reported for several self-reactive antibodies targeting voltage-gated ion channels in paraneoplastic channelopathies such as acquired neuromyotonia and Lambert–Eaton syndrome^1^. Tumour antigens are thought to trigger generation of self-reactive antibodies to tumour cell surface proteins including ion channels. Binding of these self-reactive antibodies alone is insufficient to significantly suppress channel function. Instead, long-time incubation (2∼24 h) of divalent antibodies is found to induce channel internalization, which in turn causes neuromuscular transmission defects^38^,^39^,^40^,^41^. In this study, we found that a minimum of 6 h incubation was required for Y4 to exert significant inhibition of KCNK9 conductance. Short-time perfusion of Y4 did not show detectable effects using our electrophysiology set up. This supports downregulation of cell surface channel expression as one mechanism of Y4's action, although it does not rule out potential effects on ion permeation and/or gating. An advantage of antibodies that are capable of inducing internalization compared with antibodies that act merely as blockers is that efficacious inhibition depends on the quantity and density of antigens expressed on the cell surface. This offers a mechanism-based specificity such that only tumour cells with sufficiently high channel expression are targeted by antibodies. It is possible that targeting a subset of tumour cells can limit treatment efficacy. However, the fact that Y4 is able to suppress BEN and LX22 tumour growth, as well as 410.4 metastasis, despite cell target heterogeneity, supports a balance between specificity and efficacy. Furthermore, Y4 could have anti-tumour effects independent of KCNK9 function. For example, it can be developed into antibody-drug conjugates where it facilitates tumour-specific delivery of cytotoxic payloads to kill tumour cells. In our animal models, Y4 treatment did not achieve tumour regression but rather a reduced growth rate. The effectiveness is comparable to what was observed in preclinical testing of other therapeutic antibodies^42^,^43^.

Our study serves as a proof-of-concept that ion channels are targetable by antibodies, and that this approach has therapeutic promise. The strategies described here to develop and characterize antibodies can be applied to other ion channels, providing insight into the physiological and pathological significance of this understudied class of membrane proteins.

## Methods

### Reagents

Antibodies were from the following sources: mouse anti-human growth hormone antibody (catalogue: ab9821, Abcam, 1:1,000), rabbit anti-KCNK9 antibody (catalogue: APC-044, Alomone, 1:800), goat anti-KCNK9 antibody (catalogue: sc-11317, Santa Cruz, 1:1,000), mouse anti-β-actin antibody (catalogue: ab8227, Abcam, 1:5,000), rabbit anti-cleaved caspase-3 (catalogue: 9664, Cell Signaling, 1:1,000), rabbit anti-Ki67 antibodies (catalogue: ab15580, Abcam, 1:1,000) and APC-conjugated goat anti-mouse IgG antibody (catalogue: 405308, Biolegend, 1:100). Isotype matched mouse IgG1 (mIgG1) control was from SouthernBiotech (catalogue: 0107-01). Anti-KCNK9 monoclonal antibodies were isotyped using Rapid ELISA Mouse mAb Isotyping Kit (Pierce). Transient transfection of K2P channels was performed using Fugene6 (Promega).

### Cell culture

HEK293, COS-7, BEN lung carcinoma cells (DSMZ, Germany) and MDA-MB-231 breast cancer cells (gift from Dr Sara Sukumar) were grown in DMEM medium supplemented with 10% fetal bovine serum (FBS) and 2 mM L-Glutamine (L-Gln). HEK293-KCNK9 stable cell lines were previously generated^8^ and KCNK9 expression was induced by incubation with 1 μM tetracycline for 16 h. These cells were cultured in DMEM supplemented with 10% FBS, 2 mM L-Gln, 400 μg ml^−1^ hygromycin and 15 μg ml^−1^ blasticidin. BT-549 breast cancer cells (gift from Dr Sara Sukumar) were maintained in RPMI-1640 medium supplemented with 10% FBS and 2 mM L-Gln. Syngeneic 410 (non-metastatic) and 410.4 (metastatic) murine breast cancer cells were expanded from Fulton lab stocks and cultured in DMEM supplemented with 10% FBS, 2 mM L-Gln, 0.15% w/v sodium bicarbonate and 100 μM MEM non-essential amino acids as previously described^44^. LX22 cells (patient derived by Dr Christine L. Hann) were maintained in R10 medium—RPMI1640 medium supplemented with 10% FBS, 2 mM L-Gln, 100 μg ml^−1^ penicillin/streptavidin and 20 mM HEPES. All cells were maintained at 37 °C in 5% CO_2_. None of the cell lines used is listed in the International Cell Line Authentication Committee list of misidentified cancer cell lines and all cell lines were mycoplasma negative.

### Antibody generation and purification

Murine monoclonal antibodies were generated against the first extracellular domain (M1P1 loop) of hKCNK9 (amino acids 30∼88) expressed recombinantly in mammalian expression vectors and subsequently affinity purified for immunization^45^,^46^. Y-mAbs were generated by immunizing female, 12-week-old NZB X NZW (B/W) F1 mice and H-mAbs were generated in female, 12-week-old BALB/c mice (Jackson Laboratory). Multiple rounds of limiting dilution cloning were performed and culture supernatants from hybridomas were screened by enzyme-linked immunosorbent assays (ELISA). Clones with high ELISA titres were chosen for secondary screen by flow cytometry. Selected single clones were scaled up and monoclonal antibodies were purified using protein G agarose beads (Pierce) following the manufacturer's recommendations.

### RNA isolation and quantitative real-time PCR

Total RNA from cell lines and snap-frozen tumour tissues was extracted using RNeasy Mini Kit (Qiagen) and quantitative reverse transcriptase–PCR was performed as described previously^47^. Relative expression of each gene was normalized to human18S rRNA or mouse glyceraldehyde 3-phosphate dehydrogenase RNA. Sequences of primer pairs were listed in Supplementary Table 1.

### Western blot analysis

Protein extraction and western blotting were performed as described previously^48^. Briefly, total protein was extracted using radioimmunoprecipitation assay buffer (1% Igepal, 0.5% sodium deoxycholate and 0.1% SDS in PBS) with 1 × protease and 1 × phosphatase inhibitors (Calbiochem). Aliquots of 50 μg of total protein were loaded and separated by 4–12% SDS–PAGE (Lonza). For immunoblot analyses, proteins were transferred to a nitrocellulose membrane (GE Healthcare) by wet blotting at 30∼35 V overnight at 4 °C. Membranes were incubated for 1 h in Odyssey Licor Blocking Buffer at room temperature and then overnight with primary antibodies at 4 °C. Membranes were then washed 3 × with Tris-buffered saline (TBS) with 0.1% Tween, incubated with secondary antibody at 1:10,000 for 1 h, washed 3 × with TBS with 0.1% Tween, followed by washing 2 × with TBS. Proteins were detected and quantified using the Odyssey Infrared Imager (LI-COR Biosciences). Relative expression was normalized to actin.

### Flow cytometry analysis

Single-cell suspensions were labelled with anti-KCNK9 monoclonal antibodies for 30 min at 4 °C. After washing with ice-cold PBS, cells were incubated with APC-conjugated goat anti-mIgG secondary antibody for 30 min at 4 °C. APC signal indicates KCNK9 expression and detection by KCNK9 antibodies. Cell staining was analysed using FACS Calibur flow cytometer (BD Biosciences) or sorted using FACS Aria-II flow cytometer (BD Biosciences). For transient transfection experiments, cells were co-transfected with GFP as transfection control. Flow cytometry analysis was performed on gated GFP positive cells.

### Antibody-binding characterization

Monoclonal antibody specificity was examined based on cross-reactivity to nine recombinant hK2PM1P1 subtypes in western blottings and further verified by flow cytometry analysis of cells transiently transfected with hKCNK9 and its most closely related K2P subtype hKCNK3. Affinity of binding to recombinant human and murine KCNK9M1P1 was determined using Octet system (Pall Life Sciences). Association and dissociation rates were measured in real time by incubating probes coated with Y-mAbs in the presence of hGH-hK9M1P1 or hGH-mK9M1P1 recombinant protein. *K*_D_ was estimated by deriving association (*K*_on_) and dissociation (*K*_off_) constants from real-time measurement and calculated by: *K*_D_=*K*_on_/*K*_off_.

### Tl^+^-based fluorescence assay

A fluorescence assay previously developed for chemical screen of KCNK9 modulators was adapted for monoclonal antibodies^8^,^15^. In this assay, Tl^+^ served as a surrogate ion for K^+^. Upon tetracycline induction of KCNK9 expression, cells were incubated with 0.4 mg ml^−1^ monoclonal antibodies for 0.5–72 h at 37 °C. After wash-off, cells were loaded with a Tl^+^-sensitive fluorescent dye FluxOR (Invitrogen). Upon channel activation by 2.8 mM K^+^, Tl^+^ influx yielded a fluorescence change monitored as function of time^8^. Parental HEK293 and non-induced KCNK9 cells were negative controls.

### Flow cytometry analysis of channel internalization

Tetracycline-induced KCNK9/HEK293 or BEN cells were incubated with 0.4 mg ml^−1^ Y-mAbs for 36 h at 30 °C, to induce endocytosis. KCNK9 channels remained on the plasma membrane were stained with a polyclonal anti-KCNK9 antibody followed by secondary antibody staining and flow cytometry analysis. The extent of cell surface KCNK9 reduction was quantified in two ways as follows: (1) the difference in the percentage of KCNK9-positive cells and (2) the difference in the overall fluorescence in KCNK9-positive cells by integrating fluorescence intensity and event count, reflecting the shift in the composite fluorescence intensity of KCNK9-positive cells.

### Confocal microscopic analysis of channel internalization

Y4 was labelled with Alexa Fluor 488 dye (Life Technologies). COS-7 cells were transiently transfected with hKCNK9 or hKCNK3 and mcherry was co-transfected as a transfection control. After 36 h, cells were treated with 0.4 mg ml^−1^ Y4 either for 3 h at 4 °C or for 12 h at 30 °C. At the end of incubation, cells were washed with PBS, fixed with 4% paraformaldehyde, washed with PBS and mounted with ProLong Gold Antifade Mountant containing 4,6-diamidino-2-phenylindole (Life Technologies). Images were taken at × 63/1.4 with scan zoom 1.0. For co-localization analysis, COS-7 cells were co-transfected with red fluorescent protein-tagged EEA1 (Addgene). Images were taken at × 40/1.3 with scan zoom 1.0 and quantified using Imaris software (Bitplane). A minimum threshold of red and green channels was selected. The data represent images taken from 20 different areas (*n*=20).

### Cell viability and cell death assay

Cancer cell lines were seeded in 10% heat-inactivated FBS culture medium overnight at 37 °C. Next day, cells were washed with PBS and treated with 0.4 mg ml^−1^ antibodies in 0.1 or 10% heat-inactivated FBS culture medium for 24–72 h at 37 °C. Cell viability was measured using Cell Counting Kit-8 (Dojindo Molecular Technologies). Cell death was determined by measuring LDH release using CytoTox 96 Non-Radioactive Cytotoxicity Assay (Promega) according to the manufacturer's instructions.

### Complement-dependent cytotoxicity

CDC assay protocol was adapted from previous studies^49^,^50^. Cancer cell lines were treated with 0.4 mg ml^−1^ antibodies with or without murine serum complement (Rockland) for 2 h at 37 °C. Lyophilized complement sera from mouse was reconstituted in PBS and serially diluted 1:10 to 1:1,000. Heat inactivation was performed by 30-min incubation at 56 °C. Cell death was determined as LDH release.

### ADCC assay

ADCC assay was performed following the manufacturer's recommendations^26^. Briefly, BEN target cells were seeded the day before the assay. Y4 and mIgG1 were titrated with a maximal concentration of 100 μg ml^−1^, added to BEN cells and incubated for 30 min at 37 °C. Murine Jurkat NFAT-luc+FcγRIIIa cell line was used as effector cells and seeded at a ratio of Jurkat:BEN 6:1. Co-culture was incubated for 6 h at 37 °C. Luciferase induction was measured as a readout of ADCC using Bio-Glo Luciferase assay reagent and a Tecan plate reader.

### Xenograft studies

Female, 6- to 8-week-old nu/nu or NSG mice (Charles River) were anaesthetized by i.p. injection of 5 mg kg^−1^ xylazine. Mice received 4 × 10^6^ viable BEN cells or 1 × 10^6^ viable LX22 cells (freshly dissociated from tumour xenograft) in 25 μl PBS and 25 μl Matrigel (Corning) subcutaneously in the dorsal flank. To monitor monoclonal antibodies' effects on tumour initiation, mice were randomly divided into two groups and received 4 mg kg^−1^ mIgG1 or Y4 in 100 μl PBS i.p. starting on day 0 followed by antibody injection twice a week. To monitor monoclonal antibodies' effects on tumour regression, after tumours reached 50 mm^3^, mice were randomly divided into two groups and received 4 mg kg^−1^ mIgG1 or Y4 in 100 μl PBS i.p. twice a week. Tumour volumes were estimated by measuring two dimensions (length (*a*) and width (*b*)) and calculated using the equation: *V*=*ab*^2^/2 as described previously^47^. Maximum tumour volume permitted was 2,500 mm^3^. All mice were used in accordance with guidelines from the Johns Hopkins School of Medicine Animal Care Committee.

### Immunohistochemistry

Cryostat sections were stained with anti-cleaved caspase-3 and anti-Ki67 antibodies, and counterstained with Methyl Green as previously described^48^. Proliferation and apoptotic indices were determined by computer-assisted quantification using ImageJ Software (rsb.info.nih.gov/ij/) as previously reported^48^.

### Metastasis studies

Female, 4- to 6-week-old Balb/cByJ mice (Jackson Laboratory) were randomly divided into two groups and primed by receiving 4 mg kg^−1^ mIgG1 or Y4 in 100 μl PBS i.p. twice a week for 1 week before cell injection. Viable 410.4 cells (2 × 10^5^) were injected intravenously into the lateral tail vein of mice, which were then treated with 4 mg kg^−1^ mIgG1 or Y4 in 100 μl PBS i.p. twice a week. All mice were killed on day 25 posttransplantation or earlier if moribund. Lungs were examined for surface tumour colonies under a dissecting microscope. All mice were used in accordance with guidelines from the University of Maryland Institutional Animal Care Committee.

### Cell cycle analysis

KCNK9 channels on BEN cell surfaces were labelled with Y4 and secondary antibody as described in previous section. Cells were then fixed and permeablized with 80% ethanol followed by propidium iodide staining. During flow cytometry analysis, K9^+^ and K9^−^ cells were gated and their corresponding propidium iodide signal was collected for analysis.

### Statistical analysis

Two group comparisons were analysed by two-tailed Student's *t*-test or analysis of variance. *P<*0.05 was considered significant and all data shown are mean±s.d. unless otherwise stated. Statistical analysis of co-localization was performed by calculating Pearson's coefficient of correlation between green and red channels. *r*>0.5 was considered significant.

## Additional information

**How to cite this article:** Sun, H. *et al.* A monoclonal antibody against KCNK9 K^+^ channel extracellular domain inhibits tumour growth and metastasis. *Nat. Commun.* 7:10339 doi: 10.1038/ncomms10339 (2016).

## Supplementary Material

Supplementary Information Supplementary Figures 1-8 and Supplementary Table 1
