# Supplementary material for: A monoclonal antibody against KCNK9 K+ channel extracellular domain inhibits tumour growth and metastasis
Source: Nat Commun. 2016 Feb 4;7:10339. doi: 10.1038/ncomms10339 (PMC4742836; doi:10.1038/ncomms10339)
Supplement: Supplementary Information — Supplementary Figures 1-8 and Supplementary Table 1 [file ncomms10339-s1.pdf]

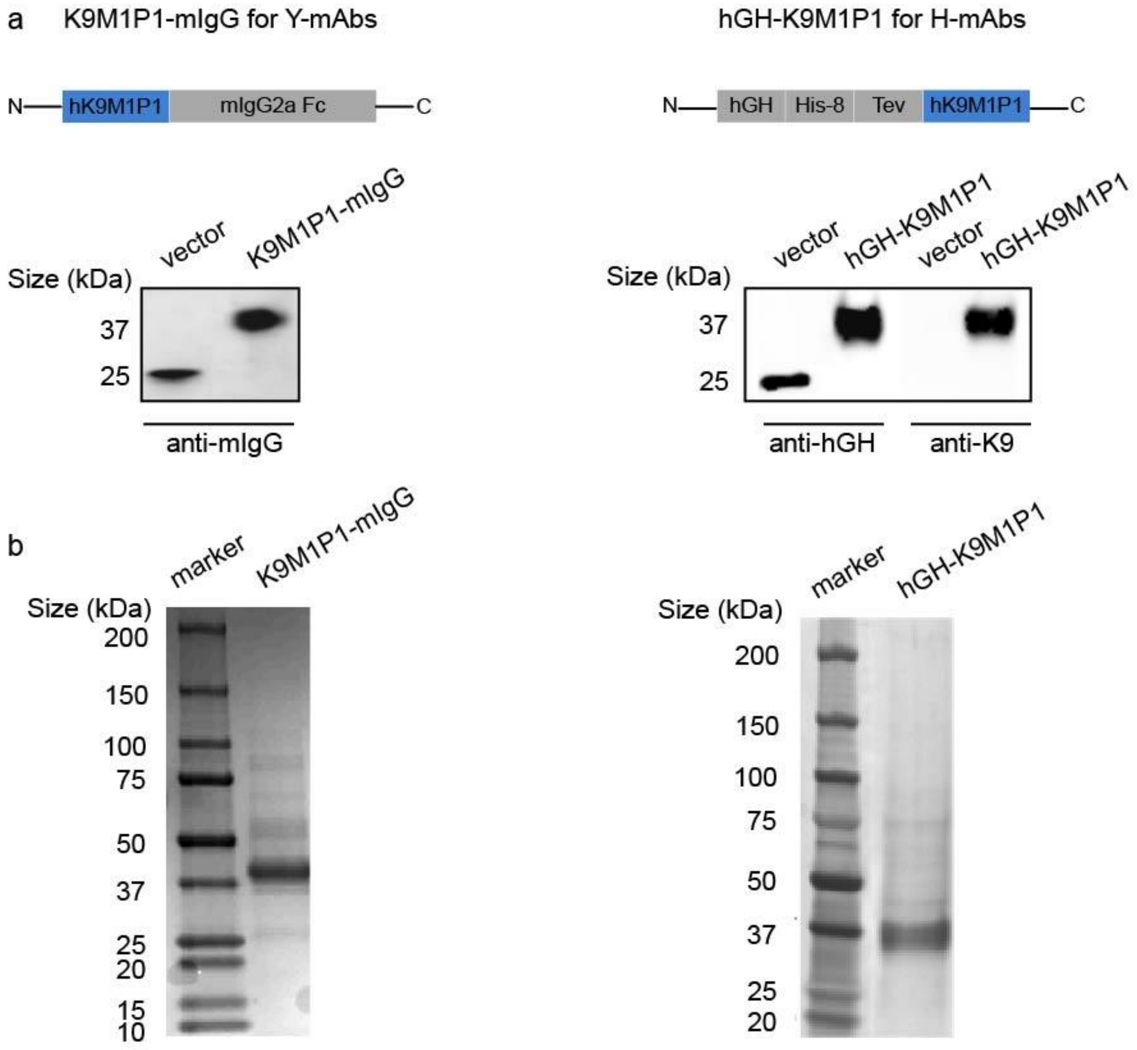

Supplementary Figure 1. Antigens generated for mAb development
(a) K9M1P1-mIgG and hGH-K9M1P1 antigen (~37 kDa) expression verified by western blot (vector: ~25 kDa).
(b) Silver staining was used to assess the purity of antigen.

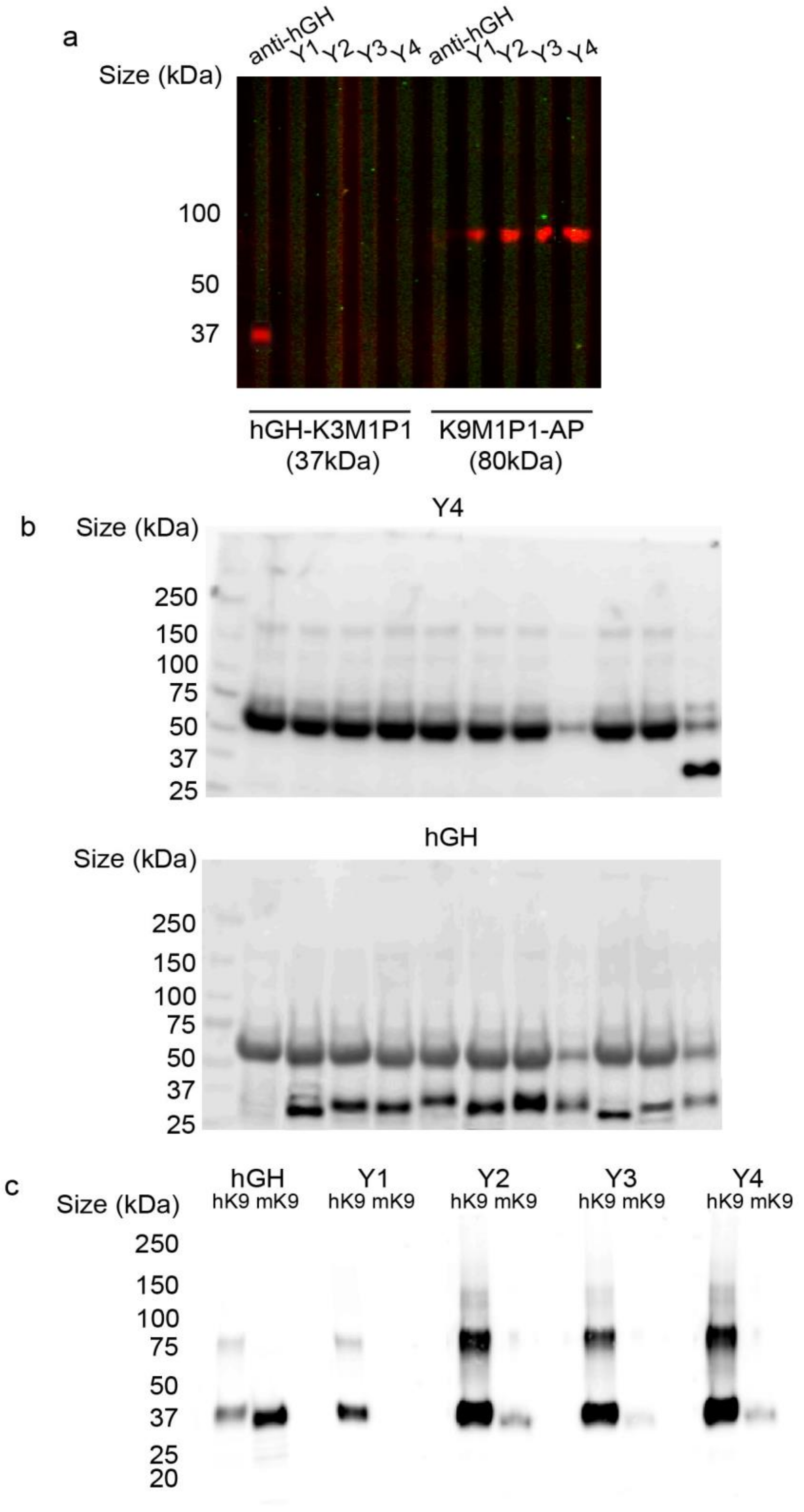

Supplementary Figure 2. Preferential binding of Y-mAbs to hKCNK9

(a) Western blot analysis of Y-mAbs against hGH-hKCNK3 and hKCNK9-alkaline phosphatase recombinant proteins. Anti-hGH antibody was used as control.

(b) Uncropped blot of of hGH-hK2PM1P1 recombinant proteins blotted with Y4 and anti-hGH antibodies.

(c) Uncropped blot of human versus murine KCNK9 recombinant proteins blotted with Y- and anti-hGH antibodies

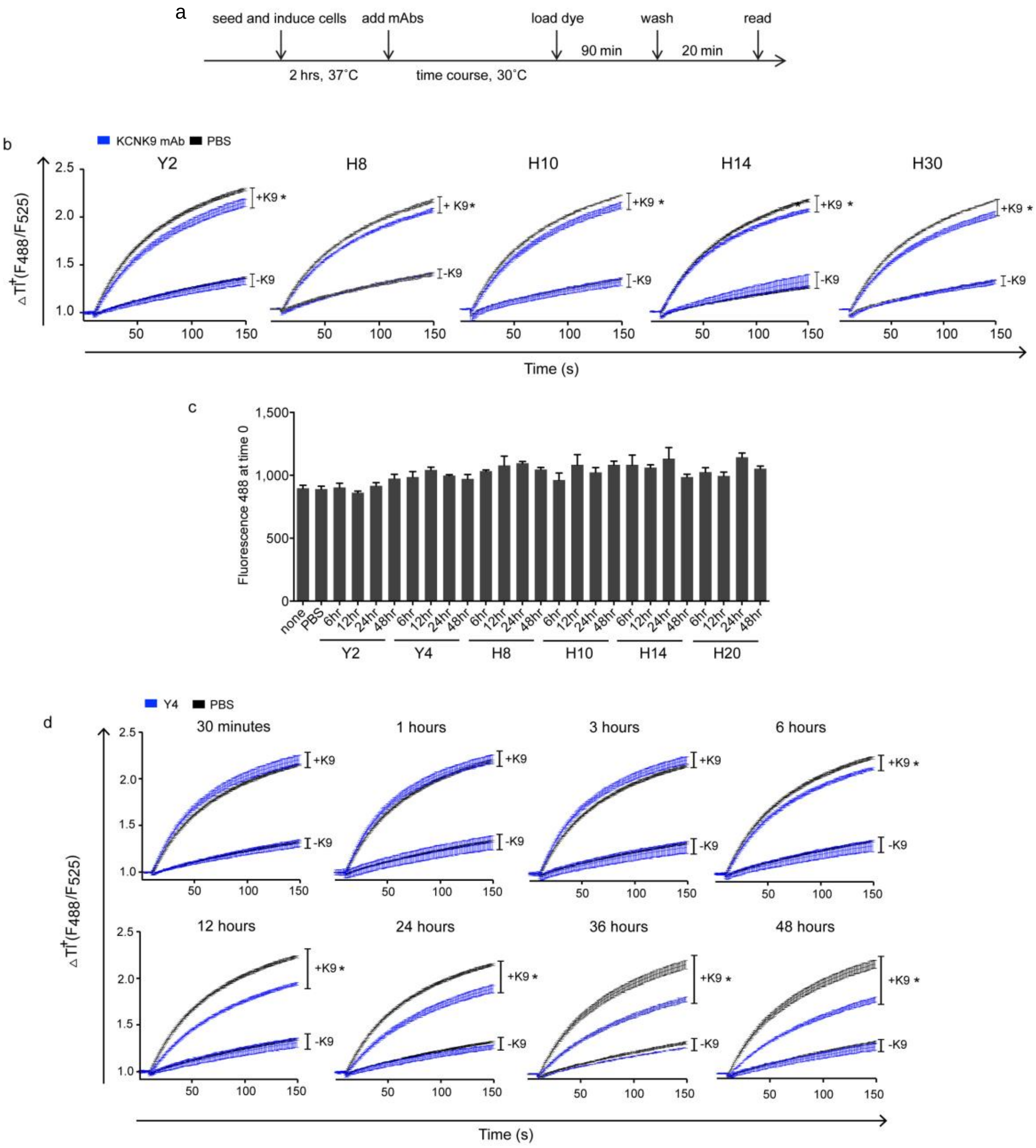

Supplementary Figure 3. MAb-mediated inhibition of Tl^+ conductance

(a) Outline of Tl^+ assay adapted for mAb screening.

(b) Representative Tl^+ traces upon 36-hour treatment of Y2, H8, H10, H14 and H30 mAbs showed marginal inhibition. mean±s.d., n=12, *p<0.05, one-way ANOVA.

(c) Fluorescence at time 0 did not show significant differences upon mAb incubation at indicated time periods suggesting no dramatic differences in cell/channel number.

(d) Representative Tl^+ traces from tetracycline-induced cells (+K9) and non-induced (-K9) cells treated with Y4 for 0.5-48 hours. mean±s.d., n=24, *p<0.01, one-way ANOVA.

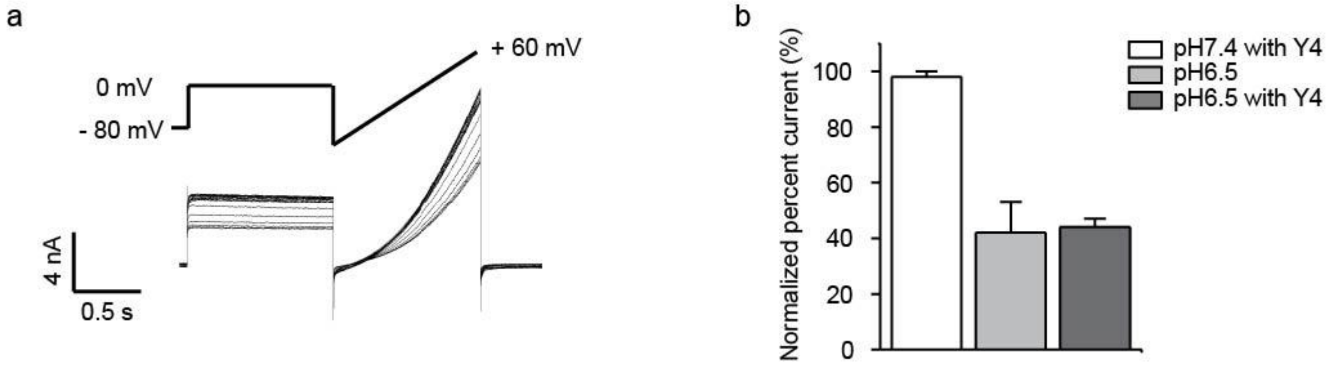

Supplementary Figure 4. Y4 showed no significant acute effects in electrophysiological recording

(a) Electrophysiological patch clamp recording protocol used to examine Y4's acute effects on hKCNK9.

(b) Quantification of hKCNK9 channel conductance in the presence of Y4. KCNK9 current was normalized to recording at pH7.4. KCNK9 extracellular domain is involved in pH sensing. Two different pH conditions were used to examine if Y4 alters channel's sensitivity to extracellular pH. mean±s.d., n=5.

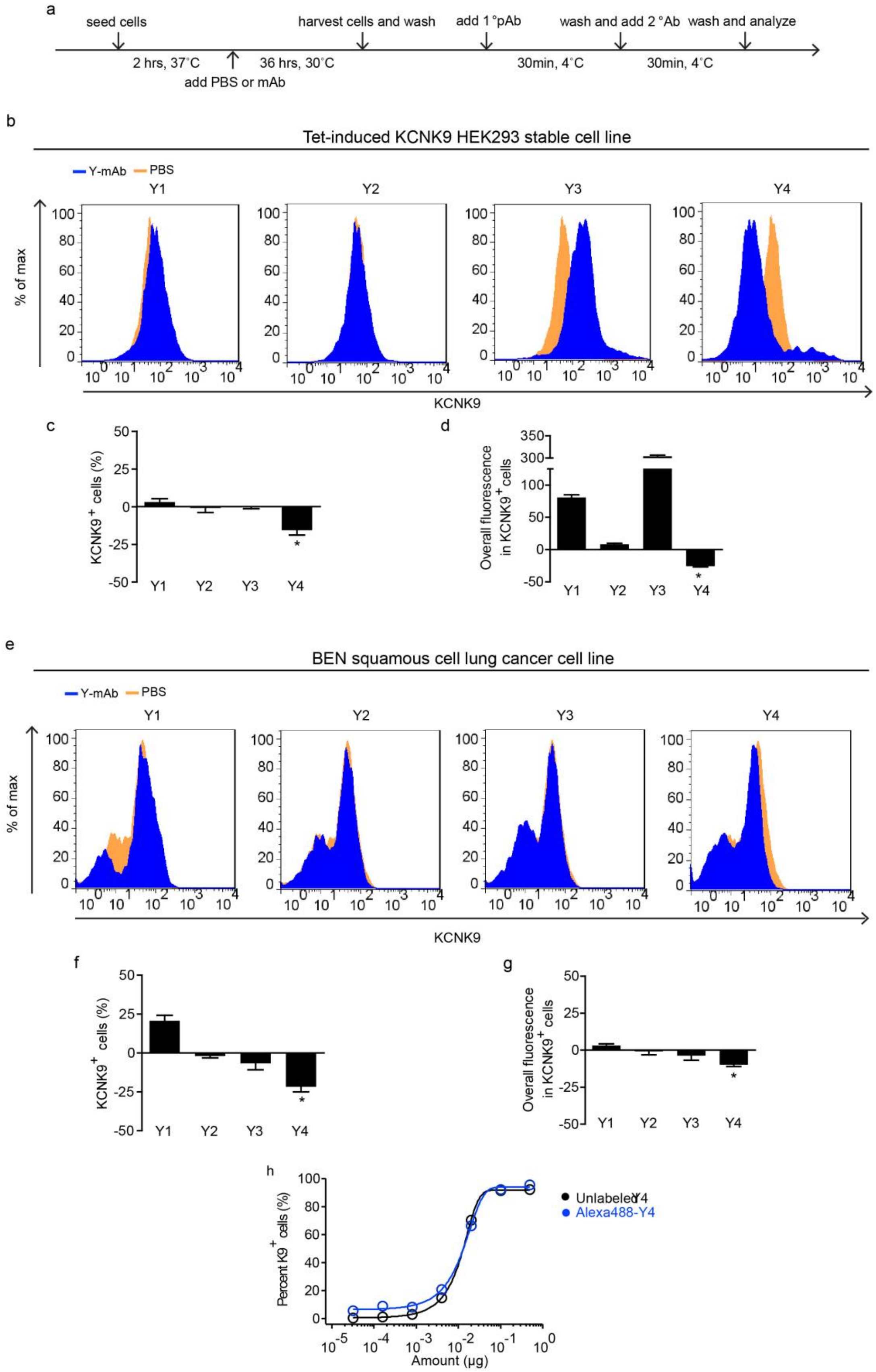
a
seed cells
harvest cells and wash
add 1 °pAb
wash and add 2 °Ab
wash and analyze
2 hrs, 37°C
add PBS or mAb
36 hrs, 30°C
30min, 4°C
30min, 4°C
b
Tet-induced KCNK9 HEK293 stable cell line
Y-mAb
PBS
Y1
Y2
Y3
Y4
% of max
KCNK9
c
KCNK9+ cells (%)
d
Overall fluorescence in KCNK9+ cells
e
BEN squamous cell lung cancer cell line
Y-mAb
PBS
Y1
Y2
Y3
Y4
% of max
KCNK9
f
KCNK9+ cells (%)
g
Overall fluorescence in KCNK9+ cells
h
Percent K9+ cells (%)
Amount (μg)
Unlabeled Y4
Alexa488-Y4

Supplementary Figure 5. Y4 reduced the number of hKCNK9 channels expressed on cell surface

(a) Outline of flow cytometry analysis set up to examine hKCNK9 remained on cell surface after Y-mAbs incubation.
(b) Representative histograms showing a significant left shift (reduction) of overall fluorescence intensity in KCNK9-positive KCNK9/HEK293 cells upon Y4 treatment.
(c) Summary of changes in the percentage of KCNK9/HEK293 cells that transitioned from KCNK9-positive to KCNK9-negative. mean±s.d., n=3, *p<0.01, two-tailed Student's t test.
(d) Summary of changes in the overall fluorescence intensity in KCNK9/HEK293 cells that remained KCNK9-positive. mean±s.d., n=3, *p<0.01, two-tailed Student's t test.
(e) Representative histograms showing a significant left shift (reduction) of overall fluorescence intensity in KCNK9-positive BEN cells upon Y4 treatment.
(f) Summary of changes in the percentage of BEN cells that transitioned from KCNK9-positive to KCNK9-negative. mean±s.d., n=3, *p<0.01, two-tailed Student's t test.
(g) Summary of changes in the overall fluorescence intensity in BEN cells that remained KCNK9-positive. mean±s.d., n=3, *p<0.01, two-tailed Student's t test.
(h) Conjugation of Y4 with Alexa488 did not alter the binding affinity of KCNK9. Tetracycline-induced KCNK9 stable cell line was stained with either unlabeled Y4 or Alexa488-labeled Y4 at indicated dilutions followed by a secondary antibody and flow cytometry analysis. Percentage of KCNK9-positive cells was plotted over mAb amount.

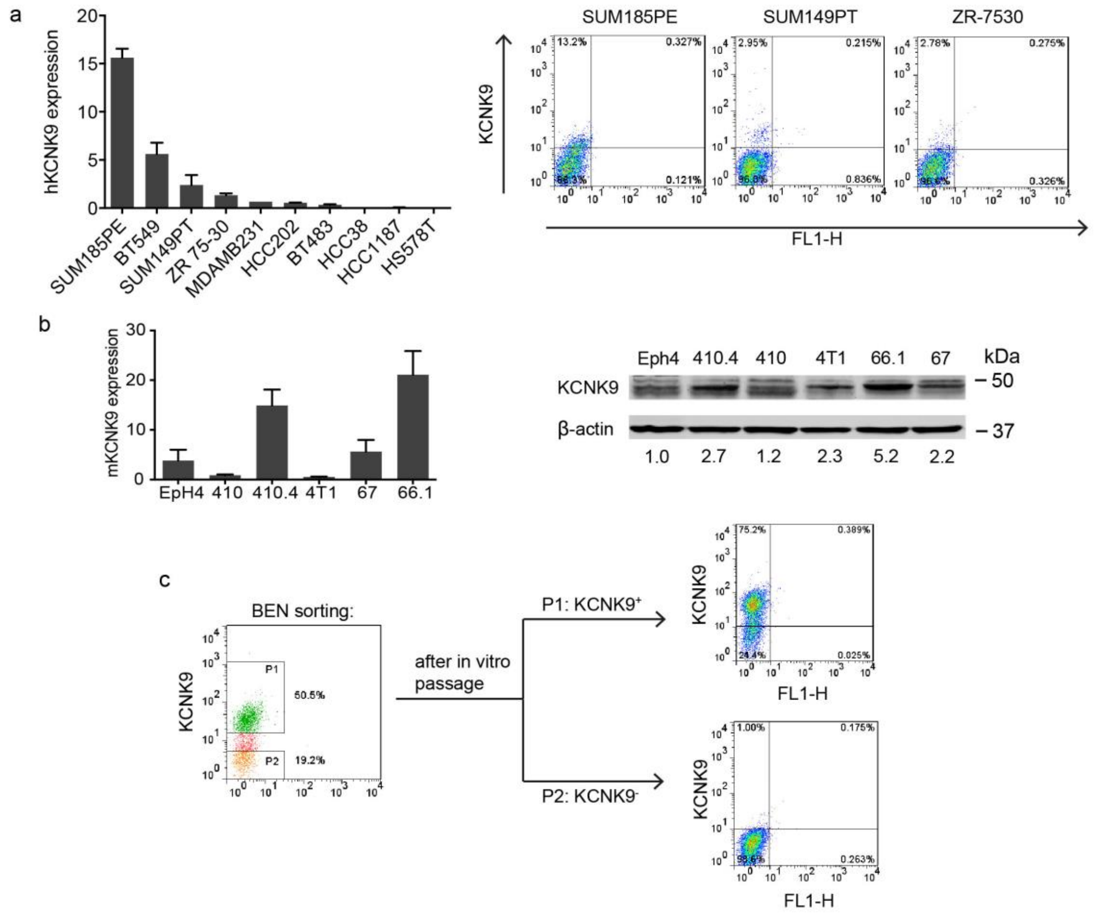

Supplementary Figure 6. KCNK9 expression in human and murine cell lines

(a) hKCNK9 expression in human breast cancer cell lines verified by qRT-PCR and flow cytometry analysis. mean±s.d., n=6.

(b) mKCNK9 expression in murine mammary cell lines verified by qRT-PCR and western blot analysis. Densitometry quantification after normalization to actin was shown below the blot. 66.1, 4T1, 410.4 are metastatic breast cancer cell lines; 67 and 410 are non-metastatic breast cancer cell lines; EpH4 is a normal mammary cell line. mean±s.d., n=6.

(c) $K9^-$ BEN cells were stable after sorting and passaging *in vitro*. BEN cells were FACS-sorted based on hKCNK9 expression and designated as P1 ($K9^+$) and P2 ($K9^-$) cells. P1 and P2 cells were isolated, separately passaged three times *in vitro*, and then re-analyzed by flow cytometry.

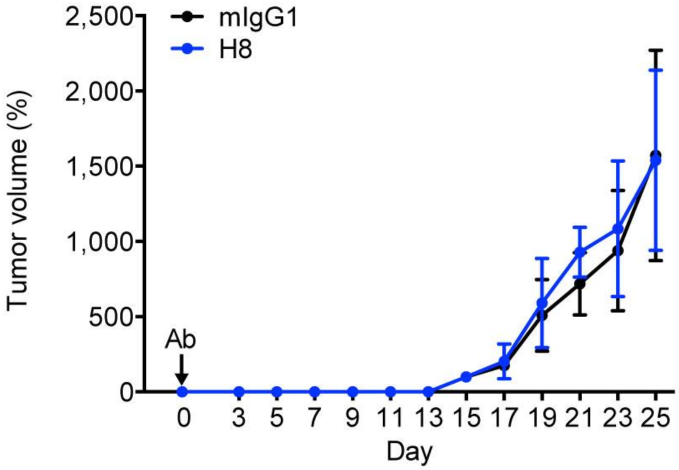

Supplementary Figure 7. H8 showed no effects in BEN xenografts

Growth curves of BEN engraftments in nu/nu mice treated with mIgG1 or H8 i.p. twice a week for 25 days. mean±s.d., n=10 per group. mAb treatment started on the same day of cell injection.

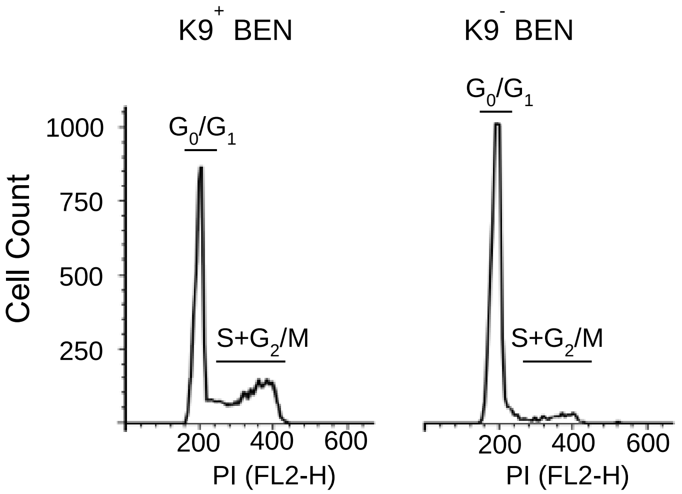

G_0/G_1	50.1%	74.6%
$S+G_2/M$	44.7%	10.9%

Supplementary Figure 8. Cell cycle analysis of $K9^+$ and $K9^-$ BEN cells
Flow cytometry analysis of BEN cells stained by Y4 to detect KCNK9 expression followed by propidium iodide (PI) staining. Cell cycle analysis was based on DNA content. G_0/G_1: 2N, S: 2N~4N, G_2/M: 4N.

Supplementary Table 1. QRT-PCR primer sequences

Gene	Accession number	Forward	Reverse
hKCNK9	NM_016601	5’-GCTCCTTCTACTTTGCGATCACG-3’	5’-CTGGAACATGACCAGTGTCAGC-3’
mKCNK9	NM_001033876	5’-CCCCTGACGCTGGTTATGTTC-3’	5’-CATGCCACAGCACTTCTTGAT-3’
mCD4	NM_013488	5’-ACACACCTGTGCAAGAAGCA-3’	5’-GCTCTTGTTGGTTGGGAATC-3’
mCD8	NM_009856	5’-CTCACCTGTGCACCCTACC-3’	5’-ATCCGGTCCCCTTCACTG-3’
mCD20	NM_026956	5’-AACCTGCTCCAAAAGTGAACC-3’	5’-CCCAGGGTAATATGGAAGAGGC-3’
mCD25	NM_008367	5’-GCGTTGCTTAGGAAACTCCTGG-3’	5’-GCATAGACTGTGTTGGCTTCTGC-3’
mCD56	NM_001081445	5’-GGTTCCGAGATGGTCAGTTGCT-3’	5’-CAAGGACTCCTGTCCAATACGG-3’
mCD68	NM_009853	5’-CCATCCTTCACGATGACACCT-3’	5’-GGCAGGGTTATGAGTGACAGTT-3’
mCD279	NM_008798	5’-CGGTTTCAAGGCATGGTCATTGG-3’	5’-TCAGAGTGTCGTCCTTGCTTCC-3’
mGranzyme B	NM_013542	5’-CCACTCTCGACCCTACATGG-3’	5’-GGCCCCCAAAGTGACATTTATT-3’
mCXCL9	NM_008599	5’-CCTAGTGATAAGGAATGCACGAG-3’	5’-CTAGGCAGGTTTGATCTCCGTTC-3’
mCXCL11	NM_019494	5’-GGCTTCCTTATGTTCAAACAGGG-3’	5’-GCCGTTACTCGGGTAAATTACA-3’
h18s rRNA	X03205	5’-GTAACCCGTTGAACCCCATT-3’	5’-CCATCCAATCGGTAGTAGCG-3’
mGAPDH	NM_008084	5’-CATCACTGCCACCCAGAAGACTG-3’	5’-ATGCCAGTGAGCTTCCCGTTCAG-3’
